# Knowledge and Attitude of Malaysian Public Towards Blood Donation During COVID-19 Pandemic: A Cross-Sectional Study

**DOI:** 10.21315/mjms-08-2024-608

**Published:** 2025-02-28

**Authors:** Siti Solehah Abdullah Muzafar Shah, Ilie Fadzilah Hashim, Zarina Thasneem Zainudeen, Intan Juliana Abd Hamid

**Affiliations:** Primary Immunodeficiency Diseases Group, Department of Clinical Medicine, Advanced Medical and Dental Institute, Universiti Sains Malaysia, Bertam, Pulau Pinang, Malaysia

**Keywords:** knowledge, attitude, blood donation, Malaysian, COVID-19

## Abstract

**Background:**

This study aimed to determine the knowledge and attitude of the Malaysian public towards blood donation during the COVID-19 pandemic.

**Method:**

This cross-sectional study utilised an online questionnaire to survey 409 Malaysians between 18 to 60 years old who were non-healthcare workers recruited via convenient snowball sampling. Data were analysed descriptively and via multiple logistic regression.

**Results:**

About half (49.2%) of the participants have good knowledge of blood donation while 71.2% of them reported a positive attitude. Gender and blood donation experience were significantly associated with knowledge of blood donation. However, only gender was associated with attitude concerning blood donation. Gender, age, income and donation experience were significantly related to the perception of blood need. No factor was identified as significantly associated with the perception of blood donation risk. The majority of the participants quoted the main reason for blood donation as to save lives.

**Conclusion:**

Most of the participants in this study showed a good knowledge and positive attitude towards blood donation. Gender, age, income and donation experience were the main associated factors. Based on these findings, future recruitment approaches for blood donors should target these identified groups, whereas promotional campaigns should be held among populations with poorer knowledge and attitudes towards blood donation, i.e., males, non-donors, younger populations and those with lower income.

## Introduction

Blood transfusion services play a vital role in the healthcare system as blood products are needed in the treatment of certain blood diseases or acute and chronic conditions that require blood as part of the treatment regime. The blood supply shortage is a common issue as the blood banks rely on the public as the main source of blood donation (1). The COVID-19 pandemic has caused a massive impact on the healthcare sector and blood bank services are not spared. There have been no reported cases of the transmission of SARS-CoV-2 through blood transfusion, and hence the risk was considered to be minimal (2). Nevertheless, a significant reduction in the supply of blood products was reported, resulting in adverse effects on blood system activities in many countries (3). The pandemic has triggered other problems such as decreasing blood donation from the public due to travel restrictions during Movement Control Order (MCO), public fear of contracting COVID-19 infection from blood donation centres, and reduced blood donation drives during the pandemic (4, 5). In Malaysia, there was a drastic drop of about 40% of the blood supply in the first few months of the COVID-19 pandemic (6).

In view of this, there is an urgent need for blood donation services to conduct assessments and respond appropriately to maintain sufficient blood donors and blood products. The relevant stakeholders involved in blood transfusion and donation services must strive to improve the knowledge and attitude of the public in raising their awareness about the importance of blood donation (7). To overcome the crisis of declining blood supplies during critical periods such as disease outbreaks, public awareness of blood donation must be prioritised to ensure continuous recruitment of blood donors. However, there is a lack of study on the knowledge and attitude of the Malaysian public on blood donation during the COVID-19 pandemic. Thus, by evaluating the public’s knowledge and attitude toward blood donation during the pandemic, the facilitators and barriers can be identified to overcome the blood supply reduction during future pandemics.

## Methods

A cross-sectional study was carried out via an online survey between March and May 2021. Inclusion criteria were Malaysians residing in the country, aged between 18–60 years old and non-healthcare workers. The sample size was estimated using the single proportion formula that gave rise to 402 participants (8). Convenient, snowball sampling was used to recruit participants via the survey link that was shared through social media such as WhatsApp, Facebook and Telegram. This study protocol was approved by the Human Research Ethics Committee USM (HREC), (USM/JEPeM/21010041). Participation in this study was voluntary and respondents’ details were ensured anonymous and confidential.

The research tool was adapted from a validated questionnaire by Ou-Yang et al. (8). The overall Cronbach’s alpha (0.73) showed an acceptable reliability. The questionnaire was in English and consisted of two sections and 34 items. Section one consisted of 10 items regarding sociodemographic characteristics. Section two contained 27 items related to the knowledge and attitude toward blood donation. However, three items were removed as they were related to the previous intervention of the study by Ou-Yang et al. (8). The reliability of the modified questionnaire was analysed, and the revised Cronbach’s alpha is 0.75, indicating good reliability across the 24 items. All items were scored on a Likert scale in which a higher score indicates more concern, positive attitudes and higher knowledge. The average time to answer the questionnaire was around 5 to 10 minutes.

Data entry and analysis were performed using Statistical Package for Social Sciences (SPSS) version 26.0. Descriptive and inferential analyses were performed. Descriptive statistics including mean (SD) for numerical variables, and frequency (percentage for categorical variables) were reported for sociodemographic characteristics. Simple and multiple logistic regressions were used to analyse the associated factors of demographic characteristics with knowledge and attitude status. The variables with *p*-value < 0.25 were selected for multivariate analysis and a forward and backward method were used for final model determination. Multiple logistic regression models contained six independent variables, i.e., gender, age, educational level, occupation, income and donation experience. Statistical significance was set at 0.05.

## Results

A total of 409 participants answered the questionnaire. The participants’ sociodemographic data were summarised in Table 1.

### Public Perceived Knowledge of Blood Donation During COVID-19 Pandemic

Table 2 outlines the perceived knowledge of blood donation among the study participants. For items Q6, Q7 and Q8, the score of 1, 2 and 3 on the Likert scale were categorised as having poor perceived knowledge, while scores 4 and 5 were deemed as good perceived knowledge. The majority of the participants reported poor perceived knowledge about blood donation policy (57.7%) and blood donation procedure (52.3%). In contrast, most of them had a good perceived knowledge of blood donation requirements (57.7%).

Table 3 shows the multiple logistic regression results for the perceived blood donation knowledge. Only female gender and donation experience were significantly associated with good perceived knowledge (*p* < 0.05). Females were more likely to have good perceived knowledge compared to males. However, the strongest predictor of having good perceived knowledge of blood donation was previous experience in donating blood. Blood donors were more likely to report a good perceived knowledge compared to those without blood donation experience.

### Public Attitude on Blood Donation During COVID-19 Pandemic

Table 4 shows the study participants’ attitudes toward blood donation during the COVID-19 pandemic. Under the domain of concern regarding COVID-19 infection, most of them reported a good attitude by mean proportion ranging from 68.0% to 77.5% participants. Similarly, most of them (74.1% to 96.1%) had a good attitude toward blood needs during the pandemic. More than half (56.2% to 62.3%) believed that they would not contract COVID-19 either on the way, during or after blood donation, and almost two-thirds (65.5%) of them felt that the staff at the blood centres would ensure their safety during blood donation.

As shown in Tables 5a–5c, there was a significant association between gender and concern about COVID-19 infection. Females were more likely to have a higher concern about COVID-19 infection during blood donation than males. Next, four independent variables showed statistically significant association with the perception of blood needs during the pandemic, including gender, age, income and donation experience. The strongest predictor of having a good perception of blood need during the pandemic was previous experience with blood donation. Blood donors were more likely to have a good perception than individuals who had never donated blood. Besides, females were more likely to report a good perception of blood needs than males. Individuals aged between 26 and 35 years old also had a better perception compared to other age groups. However, individuals from lower income groups (B40) had a lower probability of having a good perception of blood needs. On the other hand, the remaining sociodemographic characteristics were not significant predictors of the perception of the donation risk of COVID-19.

### Public Reasons for Blood Donation

**Figure 1:**
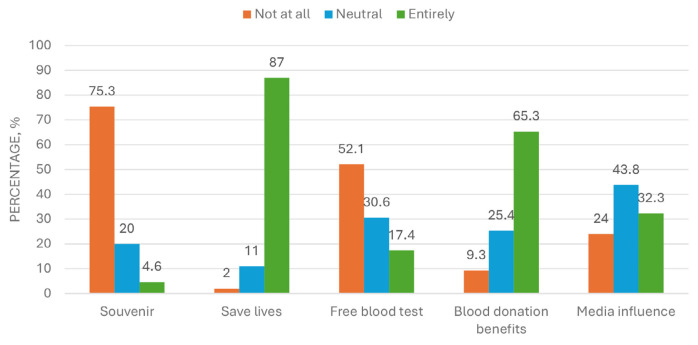
Malaysian public reasons for blood donation

Figure 1 outlines the descriptive summary of the reasons for blood donation as stated by the study participants. Most of them quoted the reason as saving lives (87.0%), followed by other benefits of blood donation (65.3%). On the other hand, almost three-quarters (75.3%) of them donated blood for the souvenirs and another half (52.1%) for the free blood test. Media influence was the lowest reason for blood donation.

## Discussion

According to the National Blood Centre, the declining blood supply in blood banks nationwide since the beginning of the COVID-19 pandemic could be attributed to the fear of being infected with COVID-19 through the blood donation process. Despite the implementation of the MCO, the Malaysian National Security Council permitted the National Blood Centre and blood banks to conduct blood donation campaigns. Blood donors were also allowed to donate blood and the safety of the blood donation process was ensured (11). However, the Malaysian public was found to demonstrate a high concern about the COVID-19 pandemic (9). Therefore, it is vital to determine the knowledge and attitude of the Malaysian public towards blood donation during the COVID-19 pandemic.

In this research, the majority of study participants were females, aged between 18 to 25 years old, single, students, and generally healthy with no underlying diseases. The age distribution was reflective of the population of Malaysia as the actual median age of the Malaysian population was 29.2 years old (10, 11). The percentage of Malaysians aged above 15 years old who used the internet has increased from 84.2% in 2019 to 89.6% in 2020, hence this could explain why the study participants of this online survey were mainly dominated by the younger population. Most of the participants had no previous experience in donating blood. According to the report by the National Blood Centre, only 0.50% or 118, 285 Malaysians have registered as new blood donors from the whole Malaysian adult population (23.6 million) in 2023 and mainly from the age of 17 to 24 years old (9). In addition, most of them also never had any family members or close friends who received a blood transfusion before or needed blood products. Hence, these factors might have led to a lower motivation to donate blood.

In previous literature, the knowledge of primary healthcare users towards blood donation was correlated with gender, educational level and previous blood donation (12). In two studies, even non-donors also demonstrated a good knowledge of blood donation (1, 13). However, a study among blood donors in Kelantan demonstrated poor knowledge regarding blood safety and permanently deferred criteria of blood donation, besides having misconceptions about the safety of blood donation (14). In contrast, university students demonstrated a good knowledge of blood donation (15). Apart from that, previous blood donors often reported a better knowledge of blood donation (16). Health science education background and mass media exposure were also associated with a higher level of knowledge (17, 18).

In this study, the majority of the participants were students. The high level of awareness among them might be a result of their voluntary participation in research revolving around blood donation. Furthermore, the potential sources of safe blood donors that meet the blood requirements are mostly young and healthy students (17). In comparison, Miah excluded healthcare workers as they were more likely to have good knowledge and attitude towards blood donation due to their higher awareness about blood donation from their working environment (7). However, in the actual setting, the knowledge of healthcare workers may vary depending on their workplaces, training countries where they did their undergraduate studies, qualification, years of service and availability of the transfusion policy (19, 20).

In the past, most studies showed that the Malaysian public has a good perception of blood needs. Even non-donors and university students demonstrated a good attitude toward blood donation despite having a poor practice of donating blood. Their attitude and practice towards blood donation were associated with education level, occupation, and field of study (13, 15). Elias et al. (16) discovered that the younger population (mean age 24.2 years old) reported high awareness, positive attitude and high intention to donate blood in the future. The level of knowledge, department, mass media exposure and social responsibility were significantly associated with a positive attitude toward blood donation among students (17). In Saudi Arabia, males were more likely to perceive blood transfusion as a high-risk procedure compared to previous blood recipients and donors who were likely to have a better perception of the benefits and overall risk of blood transfusion. In addition, the older population in the country showed a higher probability of having a negative perception of the benefits of blood transfusion (21).

In view of these findings, it is vital to raise public awareness about blood donation so that more potential blood donors can be recruited to maintain sufficient blood supply. To mitigate the risk of COVID-19, the standard donor management protocols including the practice of hand sanitisation, maintaining a safe distance between donation couches, wearing masks and temperature monitoring of both the donors and the blood bank staff must be implemented to safeguard the public confidence towards donating blood (22).

With regard to the reason for blood donation, the majority of the study participants claimed they did it to save lives. Sayed Ahmed et al. (5) found that the humanity factor was the most common motivation for first-time blood donors. This was echoed by another study that reported humanity as the main reason for donating blood among students (18). Another study in Kelantan attributed the practice of blood donation as a show of concern for the wellbeing of others (1). On the other hand, the commonest barrier to blood donation among non-donors was the refusal to give blood to other religions and races (13). Fear of needles, pain, or discomfort were the top barriers among non-donors from Hospital Universiti Sains Malaysia, Kelantan (1). Among students, concerns about sanitation and the risk of infection during donation were the barriers against blood donation (18). On a similar note, a study in Ethiopia depicted that the majority of the participants did not donate blood as they were not approached (23). More importantly, mobile blood donation vehicles were shown to be more popular than established centres (7). This finding supports the current shortage of blood donations as most mobile drives were suspended during the pandemic. To overcome a reduction in the number of blood donors, it is vital to improve the awareness of the importance of maintaining sufficient blood supply, besides providing mobile donation sites and sending regular invitations to the donors (5).

In addition, it is essential to ensure that blood donation sites are safe, pleasant and convenient to attract new donors and ensure returned donors (24). Improving knowledge of blood donation may result in better public awareness of the benefits and risks of blood donation. Nevertheless, knowledge concerning blood donation safety does not necessarily reduce the risk perception as many people still perceive blood transfusion as a risky procedure (21). To reinforce a positive attitude that facilitates blood donation practice, blood bank services should make more efforts to improve the donation experience and provide continuous medical education to allow the public to remove misconceptions about blood donation. Significant predictors identified in this study, including gender, age and donation experience should be considered during blood donation programme development.

Previously, a cross-sectional survey in Malaysia demonstrated television and the internet as the main media portals used by the Malaysian Ministry of Health and Malaysian National Security Council to disseminate important health information during the pandemic (25). The main source of information related to blood donation was from social media (13). These sources indirectly form the beliefs and perceptions of the general population, especially during the pandemic (25). This finding showed that rigorous efforts should be taken to select the best media to educate the public and raise their awareness about blood donation. Furthermore, different communication methods to motivate blood donors have also been studied previously with text messages being the most efficient reminder system, followed by emails. About 1.5% of the blood donors would donate blood within seven days after they receive the reminder. Furthermore, the time of blood donation campaigns can also affect the turnout rate of the donors (7). Ou-Yang et al. (8) stated that the number of blood donors significantly increased with a systematic communication measure. Both first-time and repeated donors perceived the same level of blood needs and donated blood to save lives. In addition, there was a greater intention to donate blood during the pandemic as blood donors perceived a higher level of blood needs and a lower level of COVID-19 infection risk related to blood donation (8).

Previously, a study involving Malaysian adults aged 18 to 60 years in Penang identified lifestyle-related factors such as working hours, family history and fear of the donation process as significant factors in blood donation. This study also suggested donor awareness programmes should focus on females, single, and individuals from lower socioeconomic backgrounds. In addition, donor retention strategies should be aimed at those with a previous family history of blood recipients (26). Based on these findings, blood banks have devised a targeted approach with several response plans, including call-outs for healthy repeat donors to visit the blood bank and donate blood whenever they are free. Proactive communication initiatives by blood banks including printed, electronic, and social media were also taken to ensure good public awareness about the donation process safety and blood needs (22).

A recent study utilising phone calls and interviews among blood donors found that the most common reason behind their lapse in blood donation was that they chose to stay home to avoid being infected with COVID-19. However, about 63.4% of the participants would return to donate if they received regular invitations while another 52.4% would like to return if the pandemic was under control (5).

Positive experiences of blood donation in the form of perceived support from peers and competent phlebotomists can also motivate repeated blood donation. However, individuals with negative experiences such as encountering incompetent phlebotomists and unappreciative blood bank staff would require certain mechanisms to convert their negative feelings into positive experiences (27). For instance, Mohammed and Essel suggested that public education that encourages regular donors to donate during blood supply shortages and the kind attitude of blood bank staff can potentially motivate donors and eliminate barriers to blood donation (28).

Most of the study participants with blood donation experience had a good knowledge of blood donation. They were also willing to donate in the future without expecting any post-donation reward. This finding suggests the need to educate young people on the importance of blood donation in saving lives and the relevant requirements for blood donation (16). Sharing information on appropriate public health measures taken in the community to ensure a safe environment for donors can maintain public confidence and motivate them to continue donating blood (4).

There were a few limitations to this study. The findings may not be inferred from the general Malaysian population because of the convenient sampling that resulted in mostly young participants. Other limitations included a short period of data collection, the use of the English language in the questionnaire, and the online distribution of the questionnaire due to the restrictions from the pandemic. Thus, the survey might not have reached the majority of the population and the lack of face-to-face interaction might also result in some misinterpretation of the questions. Future studies should consider adding more specific questions about factors that can lead to declined blood donation and shortage of blood supplies. Besides that, it is recommended to increase the number of respondents and broaden the target to a more diverse population in the country to gather a more representative sample.

## Conclusion

In summary, this study reported a good level of perceived knowledge and attitude toward blood donation during the COVID-19 pandemic. There was generally a sufficient level of awareness of blood donation during the pandemic. However, reduced blood donation and temporary suspension of blood collection facilities have significantly affected the supply of blood products during the pandemic. Appropriate risk assessment and management must be taken to prevent recurrence. Future research is warranted to determine the specific facilitators and barriers influencing blood donation among Malaysians. Educational campaigns to promote blood donation should target male gender, non-donors, and lower income populations. Furthermore, blood services must adopt a rational approach to ensure public confidence in blood safety by weighing the situation of the extent of COVID-19 spread, the level of community cases transmission, local risk of transfusion transmission. By doing so, the quality of the health care system will be safeguarded without compromising the operational impacts and cost-effectiveness of the interventions.

## Figures and Tables

**Table 1:** Participants’ sociodemographic characteristics (*n* = 409)

Variables	*n*	%
Consented	409	100.0

**Gender**		
Male	98	24.0
Female	311	76.0

**Age**		
18–25 years old	219	53.5
26–35 years old	107	26.2
36–45 years old	41	10.0
46–60 years old	42	10.3

**Marital status**		
Married	132	32.3
Unmarried	277	67.7

**Education level**		
No formal education	2	0.5
Primary school	3	0.7
Secondary school	34	8.3
College or university	370	90.5

**Occupation**		
Government	87	21.3
Private	73	17.8
Self-employed	20	4.9
Student	202	49.4
Unemployed	27	6.6

**Income**		
No income	194	47.4
B40	146	35.7
M40	62	15.2
T20	7	1.7

**Documented illnesses**		
Yes	49	12.0
No	360	88.0

**Donation experience**		
Never	260	63.6
1–5 times	96	23.5
6–10 times	34	8.3
More than 10 times	19	4.6

**Current state**		
Kuala Lumpur	27	6.6
Johor	28	6.8
Kedah	52	12.7
Kelantan	29	7.1
Malacca	8	2.0
Negeri Sembilan	6	1.5
Pahang	31	7.6
Penang	67	16.4
Perak	25	6.1
Perlis	7	1.7
Sabah	18	4.4
Sarawak	14	3.4
Selangor	76	18.6
Terengganu	21	5.1

**Have any of your family members or close friends ever received a blood transfusion or been waiting for blood during the pandemic?**		
Yes	46	11.2
No	363	88.8

**Table 2:** Malaysian perceived knowledge on blood donation (*n* = 409)

Items	Description	Score			

		Poor	%	Good	%
Q6	What do you know about the blood donation policy?	236	57.7	173	42.3
Q7	What do you know about the blood donation procedure?	214	52.3	195	47.7
Q8	What do you know about the blood donation requirements?	173	42.3	236	57.7

**Table 3:** Logistic regression analysis of Malaysian perceived knowledge on blood donation

	Unadjusted model			Adjusted model		

	ß	Sig.	Odds ratio	ß	Sig.	Odds ratio
**Gender**						
Male	0		1	0		1
Female	0.28	0.21	1.33 (0.84, 2.10)	0.87	0.002*	2.40 (1.36, 4.24)

**Age**						
18–25 years old	0		1	0		1
26–35 years old	0.68	0.004	1.98 (1.23, 3.18)	−0.39	0.41	0.67 (0.25, 1.75)
36–45 years old	0.65	0.05	1.92 (0.97, 3.81)	−0.11	0.85	0.89 (0.27, 2.94)
46–60 years old	−0.17	0.61	0.83 (0.42, 1.64)	−0.67	0.27	0.50 (0.15, 1.69)

**Education level**						
Without tertiary education	0		1	0		1
Tertiary education	0.90	0.01	2.48 (1.21, 5.04)	0.48	0.23	1.62 (0.73, 3.60)

**Occupation**						
Government	0		1	0		1
Private	0.06	0.83	1.07 (0.56, 2.01)	−0.22	0.57	0.79 (0.35, 1.76)
Self-employed	−0.34	0.48	0.70 (0.26, 1.87)	−0.60	0.31	0.54 (0.16, 1.77)
Student	−0.58	0.02	0.55 (0.33, 0.92)	−0.58	0.30	0.55 (0.18, 1.69)
Unemployed	−0.57	0.19	0.56 (0.23, 1.34)	−0.72	0.25	0.48 (0.13, 1.69)

**Income**						
No income	0		1	0		1
B40	0.63	0.004	1.89 (1.22, 2.92)	0.13	0.71	1.14 (0.54, 2.40)
M40	0.18	0.52	1.20 (0.67, 2.13)	−0.63	0.25	0.52 (0.17, 1.56)
T20	0.53	0.49	1.70 (0.37, 7.84)	0.32	0.75	1.37 (0.18, 10.44)

**Donation experience**						
Never	0		1	0		1
Yes	1.67	0.000	5.34 (3.40, 8.38)	1.99	0.000*	7.33 (4.15, 12.97)

Note:

* indicates *p*-value < 0.05

**Table 4:** Malaysian attitude on blood donation (*n* = 409)

Items	Description	Score			

		Poor	%	Good	%
**Concern on COVID-19 infection**					
Q1	How fearful do you feel about the COVID-19 pandemic?	100	24.4	309	75.6
Q2	How anxious do you feel about the COVID-19 pandemic?	115	28.1	294	71.9
Q3	How worried do you feel about the COVID-19 pandemic?	92	22.5	317	77.5
Q4	How worried do you feel about infecting COVID-19?	69	16.9	340	83.1
Q5	How much impact does COVID-19 have on you?	131	32.0	278	68.0

**Perception on blood need**					
Q9	What do you think is the health impact of blood donation?	103	25.2	306	74.8
Q10	Do you agree that the blood you donated will be used to patient who really needs it?	16	3.9	393	96.1
Q12	How serious do you think the blood shortage is in Malaysia?	106	25.9	303	74.1
Q13	How important do you think blood donation is during the COVID-19 pandemic in Malaysia?	57	13.9	352	86.1
Q14	How urgent do you think blood donation is during the COVID-19 pandemic in Malaysia?	62	15.2	347	84.8
Q15	How much lost will there be if no one donates blood during the pandemic?	46	11.2	363	88.8

**Donation risk of COVID-19**					
Q16	How likely do you think you will get infected with COVID-19 on the way to donate blood?	248	60.6	161	39.4
Q17	How likely do you think you will get infected with COVID-19 at the donation site when you donate blood?	255	62.3	154	37.7
Q18	How likely do you think that the staff at the blood centre can ensure your safety during donation?	141	34.5	268	65.5
Q19	How likely do you think you will get infected with COVID-19 after blood donation?	230	56.2	179	43.8

**Table 5a:** Multiple logistic regression of Malaysian attitude towards blood donation during COVID-19 pandemic (concern on COVID-19 infection)

Domain	Concern on COVID-19 infection					

	Unadjusted model			Adjusted model		

	ß	Sig.	Odd ratio (95% CI)	ß	Sig.	Odd ratio (95% CI)
**Gender**						
Male	0		1	0		1
Female	1.37	0.000*	3.97 (2.38, 6.61)	1.40	0.000*	4.07 (2.32, 7.13)

**Age**						
18–25 years old	0		1	0		1
26–35 years old	0.42	0.17	1.52 (0.83, 2.80)	0.88	0.11	2.42 (2.32, 7.13)
36–45 years old	−0.47	0.20	0.62 (0.29, 1.28)	0.04	0.93	1.05 (0.29, 3.76)
46–60 years old	0.36	0.41	1.44 (0.60, 3.44)	1.29	0.07	3.63 (0.89, 14.67)

**Education level**						
Without tertiary education	0		1	0		1
Tertiary education	0.71	0.05	2.03 (0.99, 4.15)	0.57	0.17	1.77 (0.77, 4.09)

**Occupation**						
Government	0		1			
Private	−0.14	0.71	0.86 (0.40, 1.86)			
Self-employed	−0.31	0.58	0.72 (0.23, 2.28)			
Student	−0.07	0.80	0.92 (0.49, 1.73)			
Unemployed	−0.16	0.76	0.85 (0.29, 2.43)			

**Income**						
No income	0		1	0		1
B40	0.47	0.09	1.61 (0.92, 2.81)	0.86	0.09	2.37 (0.86, 6.53)
M40	−0.22	0.50	0.79 (0.41, 1.53)	0.13	0.83	1.13 (0.32, 3.99)
T20	0.59	0.58	1.81 (0.21, 15.44)	0.70	0.58	2.02 (0.16, 24.70)

**Donation experience**						
Never	0		1			
Yes	−0.22	0.33	0.79 (0.48, 1.29)			

Note:

* indicates *p*-value < 0.05

**Table 5b:** Mltiple logistic regression of Malaysian attitude towards blood donatin during COVID-19 pandemic (perception on blood need)

Domain	Perception on blood need					

	Unadjusted model			Adjusted model		

	ß	Sig.	Odd ratio (95% CI)	ß	Sig.	Odd ratio (95% CI)
**Gender**						
Male	0		1	0		1
Female	0.94	0.01	2.57 (1.17, 5.64)	1.73	0.000*	5.67 (2.23, 14.4)

**Age**						
18–25 years old	0		1	0		1
26–35 years old	1.13	0.07	3.10 (0.89, 10.7)	1.96	0.039*	7.13 (1.10, 45.8)
36–45 years old	−0.18	0.74	0.82 (0.26, 2.58)	0.35	0.71	1.43 (0.20, 10.08)
46–60 years old	0.15	0.81	1.16 (0.32, 4.14)	0.49	0.62	1.64 (0.22, 12.21)

**Education level**						
Without tertiary education	0		1			
Tertiary education	0.49	0.38	1.64 (0.54, 5.02)			
Occupation						
Government	0		1			
Private	0.24	0.71	1.27 (0.34, 4.71)			
Self-employed	−0.86	0.25	0.41 (0.09, 1.84)			
Student	−0.07	0.87	0.92 (0.34, 2.46)			
Unemployed	0	–	–			

**Income**						
No income	0		1	0		1
B40	0.47	0.09	1.61 (0.92, 2.81)	0.86	0.09	2.37 (0.86, 6.53)
M40	−0.22	0.50	0.79 (0.41, 1.53)	0.13	0.83	1.13 (0.32, 3.99)
T20	0.59	0.58	1.81 (0.21, 15.44)	0.70	0.58	2.02 (0.16, 24.70)

**Donation experience**						
Never	0		1	0		1
Yes	1.64	0.008	5.17 (1.53, 17.45)	2.66	0.000*	14.40 (3.21, 64.47)

Note:

* indicates *p*-value < 0.05

**Table 5c:** Multiple logistic regression of Malaysian attitude towards blood donation during COVID-19 pandemic (perception on donation risk of COVID-19)

Domain	Concern on COVID-19 infection					

	Unadjusted model			Adjusted model		

	ß	Sig.	Odd ratio (95% CI)	ß	Sig.	Odd ratio (95% CI)
**Gender**						
Male	0		1			
Female	−0.19	0.39	0.82 (0.51, 1.29)			

**Age**						
18–25 years old	0		1	0		1
26–35 years old	0.21	0.36	1.23 (0.77, 1.96)	−0.19	0.65	0.82 (0.36, 1.88)
36–45 years old	0.39	0.25	1.47 (0.75, 2.90)	−0.13	0.79	0.87 (0.31, 2.40)
46–60 years old	0.04	0.89	1.04 (0.54, 2.02)	−0.47	0.34	0.61 (0.22, 1.67)

**Education level**						
Without tertiary education	0		1			
Tertiary education	0.11	0.73	1.12 (0.58, 2.17)			

**Occupation**						
Government	0		1	0		1
Private	−0.16	0.60	0.84 (0.45, 1.58)	−0.20	0.57	0.81 (0.40,1.65)
Self-employed	0.10	0.83	1.11 (0.41, 2.98)	0.13	0.80	1.14 (0.39, 3.33)
Student	−0.40	0.12	0.67 (0.40, 1.11)	−0.82	0.11	0.43 (0.15, 1.21)
Unemployed	−0.37	0.39	0.68 (0.28, 1.63)	−0.63	0.26	0.52 (0.17, 1.61)

**Income**						
No income	0		1			
B40	0.07	0.73	1.07 (0.70, 1.65)			
M40	0.21	0.46	1.23 (0.69, 2.20)			
T20	0.93	0.27	2.55 (0.48, 13.4)			

**Donation experience**						
Never	0		1			
Yes	0.06	0.75	1.06 (0.71, 1.59)			

Note:

* indicates *p*-value < 0.05
